# Reliable Tilt-depth estimates based on the stable computation of the tilt angle using robust vertical derivatives

**DOI:** 10.1038/s41598-024-57314-5

**Published:** 2024-03-28

**Authors:** Kamal Abdelrahman, Luan Thanh Pham, Saulo Pomponet Oliveira, Van-Hao Duong, Thong Kieu Duy, David Gomez-Ortiz, Mohammed S. Fnais, Ahmed M. Eldosouky

**Affiliations:** 1 Department of Geology and Geophysics, College of Science, King Saud University, P.O. Box 2455, 11451 Riyadh, Saudi Arabia; 2 University of Science, Vietnam National University Hanoi, 334 Nguyen Trai, Thanh Xuan, Hanoi, Vietnam; 3 Department of Mathematics, Federal University of Parana, Curitiba, PR Brazil; 4 VNU School of Interdisciplinary Studies, Vietnam National University, Hanoi, Vietnam; 5 Hanoi University of Mining and Geology, Hanoi, Vietnam; 6 Department of Biology and Geology, Physics and Inorganic Chemistry, ESCET, Universidad Rey Juan Carlos, Móstoles, Madrid, Spain; 7 Research Group ‘Geofísica y Geoquímica Ambiental’, Universidad Rey Juan Carlos, Madrid, Spain; 8 Department of Geology, Faculty of Science, Suez University, P.O. Box 43221, Suez, Egypt

**Keywords:** Geomagnetism, Geophysics

## Abstract

The Tilt-depth is a popular approach for determining depths of magnetic sources. As this method is based on the distance between contour levels of the tilt angle, it can lead to inaccurate depth estimates when the contour lines are distorted by the presence of noise. In this paper, we stabilize the Tilt-depth method based on the computation of stable vertical derivative obtained by the β-VDR method. The presented method is demonstrated on synthetic magnetic anomalies and real magnetic data from the Arabian Shield. The results obtained from the synthetic examples coincide well with the actual depths. These results proved the utility of the presented method in cases where the field is corrupted by noise. The real example shows that the presented method can provide valuable information on subsurface structures of the area where the Tilt-depth estimates are consistent with the result of the horizontal tilt angle. The findings show that the presented method is less sensitive to noise and can provide source edges and depths more clearly and with higher accuracy.

## Introduction

Magnetic exploration plays a key role in mapping subsurface geological structures^1^. Magnetic data interpretation is commonly used in economic resource exploration due to the magnetization contrast between surrounding rocks and ore/mineral deposits. Various magnetic interpretation methods have been introduced and applied in regional studies^2–6^ and local studies^7–10^ so far. Among these methods, the Euler deconvolution is one of the most often used methods for estimating the depth of magnetic sources. The method was first proposed by Thompson^11^ for profiles and generalized for grid data by Reid et al.^12^. The disadvantage of this technique is that it tends to yield many false solutions. Improvements in the Euler deconvolution method have made it possible to detect both the location and structural index^13–17^. However, the improved methods use high-order derivatives of magnetic data and require careful data filtering. Some authors used the analytic signal methods^18,19^ that do not require window size, but they are sensitive to noise. Thurston and Smith^20^ introduced the source parameter imaging (SPI) method that is based on second-order derivatives of magnetic data and uses a term known as the local wavenumber to provide a rapid calculation of the depth of magnetic sources. However, the method is also sensitive to noise. Smith et al.^21^ and Thurston et al.^22^ improved the local wavenumber method to determine both the depth and nature of the sources. Since the improved local wavenumber methods are based on third-order derivatives, they are more sensitive to noise. Salem and Smith^23^ introduced the normalized local wavenumber that does not depend on the nature of the source. However, their approach requires the peaks of the wavenumber profile that are difficult to detect in the presence of noise. Some authors used inversion techniques based on the Parker method to map magnetic basements^24–27^. Although these techniques can perform rapid computation for large datasets, they require average depth, magnetization, and low pass filter.

Another approach based on the Tilt angle or Tilt derivative (TDR) filter, known as the Tilt-depth method, was first introduced by Salem et al.^28,29^ can estimate the depth of sources from magnetic data without any assumptions about the window size. Other advantages of this method in comparison with the inversion techniques are that it does not depend on an initial depth, magnetization, or low pass filter^25^. Oruc^30^ also introduced the gravity Tilt-depth method that is based on the second-order derivatives of gravity data. Recently, the application of the Tilt-depth to magnetic datasets has shown great success in estimating basement depth^31–35^. The Tilt-depth method is based on derivatives of magnetic data where the vertical derivative usually computes in the frequency domain. It is well known that the vertical derivative computed by using the FFT technique is dominated by high-frequency noise^36^. For this reason, the Tilt-depth method is sensitive to noise when performing the vertical derivative calculation in the frequency domain.

Recently, the computation of vertical derivatives using upward-continued data has been addressed by several authors. Tran and Nguyen^36^ introduced high-order finite-difference formulas for vertical derivatives, and used them to calculate the downward continuation according to Taylor series expansion. In 2022, Oliveira and Pham^37^ proposed the β-VDR method using another finite-difference formula that can provide a more stable approximation of the vertical derivative of potential field data. This method was used to improve the computation of edge enhancement techniques^37^.

In this study, we introduce an improved Tilt-depth approach that uses the vertical derivative calculated by using the β-VDR method^37^instead of those from the usual frequency domain technique to provide more stable results for the depth of magnetic sources. To our knowledge, the improvement of Tilt depth method by regularized derivative methods such as the β-VDR method has not been addressed in the literature. We have found that this approach is also helpful in reducing the number of spurious solutions, and improves the accuracy of the meaningful solution. We demonstrate the applicability of the improved Tilt-depth on both synthetic examples and a real dataset of the Arabian Shield in mapping subsurface geological structures. The obtained results are compared with the assumed parameters in the case of the synthetic examples and with the horizontal tilt angle (TDX)^38^ in the case of the field example.

## Methods

The TDR is one of the most used methods for enhancing the presence of lineaments and geological contacts. The method was first introduced by Miller and Singh^39^. It is defined by the arc tangent of the ratio between the vertical derivative and the gradient horizontal:

1 $${\text{TDR}}={\text{atan}}\frac{\frac{\partial {\text{F}}}{\partial {\text{z}}}}{\sqrt{{\left(\frac{\partial {\text{F}}}{\partial {\text{x}}}\right)}^{2}+{\left(\frac{\partial {\text{F}}}{\partial {\text{y}}}\right)}^{2}}}.$$

where F is the magnetic anomaly. The horizontal derivatives in Eq. (1) can be easily estimated using the finite difference method, while the computation procedure of the vertical derivative usually performs in the frequency domain using the fast Fourier transform (FFT)^40^:

2 $$\frac{\partial {\text{F}}}{\partial {\text{z}}}=IFFT[ \left|k\right|FFT[\mathrm{F }]]$$

where $IFFT$ is the inverse Fourier transform and *k* is the wavenumber that is given as:

3 $$k=\sqrt{{k}_{x}^{2}+{k}_{y}^{2}},$$

where *k*_*x*_ and *k*_*y*_ are the wavenumbers in the x and y directions, respectively.

To map the depth to magnetic contacts, Salem et al.^28^ introduced the Tilt-depth technique that is based on the relationship between tilt angle, location and position of a contact as:

4 $${\text{TDR}}=atan\frac{{\text{h}}}{{{\text{z}}}_{{\text{c}}}},$$

where ${{\text{z}}}_{{\text{c}}}$ is the contact depth and h is the horizontal location. Equation (4) shows that the contact location (h = 0) relates to the zero values of the TDR and the depth relates to the horizontal distance between the TDR contour levels of 0 and ± $\uppi /4$.

In general, the TDR uses the vertical derivative of magnetic anomaly calculated in the frequency domain^40^. However, this approach provides unstable values^41^. To solve this issue, we suggest using the vertical derivative obtained from the β-VDR method^37^. Our method is referred to as the β -VDR Tilt-depth where the β -VDR vertical derivative of the magnetic field is defined by the following Eq. ^37^:

5 $$\frac{\partial F}{\partial z}=\frac{{c}_{1}F\left({h}_{1}\right)+{c}_{2}F\left({h}_{2}\right)+{c}_{3}F\left({h}_{3}\right)+{c}_{4}F\left({h}_{4}\right)+{c}_{5}F\left({h}_{5}\right)}{\Delta h}$$

where c_1_,…,c_5_ are given by:

6 $$\left\{\begin{array}{c}{c}_{1}=\left(2{\beta }^{3}+15{\beta }^{2}+35\beta +25\right)/12,\\ {c}_{2}=\left(-8{\beta }^{3}-54{\beta }^{2}-104\beta -48\right)/12,\\ \begin{array}{c}{c}_{3}=\left(12{\beta }^{3}+72{\beta }^{2}+114\beta +36\right)/12,\\ \begin{array}{c}{c}_{4}=\left(-8{\beta }^{3}-42{\beta }^{2}-56\beta -16\right)/12,\\ {c}_{5}=\left(2{\beta }^{3}+9{\beta }^{2}+11\beta +3\right)/12,\end{array}\end{array}\end{array}\right.$$

and $F({h}_{i})$ is the anomaly upward-continued to ${h}_{i}={z}_{0}-\beta \Delta h-\left(i-1\right)\Delta h$ with ${z}_{0}$ is the height of the observation plane, $\Delta h$ is smaller than the grid spacing and $\beta$ is a user-defined stabilizing parameter. Here, we use $\Delta h=\frac{1}{10}$ of grid spacing and $\beta$ = 30 for computing the vertical derivative^37,42^.

## Results

The applicability of the presented method in magnetic interpretation is demonstrated on both synthetic and real-world magnetic anomalies. The real field magnetic data pertains to the Arabian Shield. We also compared the proposed method with the Tilt-depth technique using the vertical derivative calculated in the frequency domain.

### Theoretical examples

**Figure 1:**
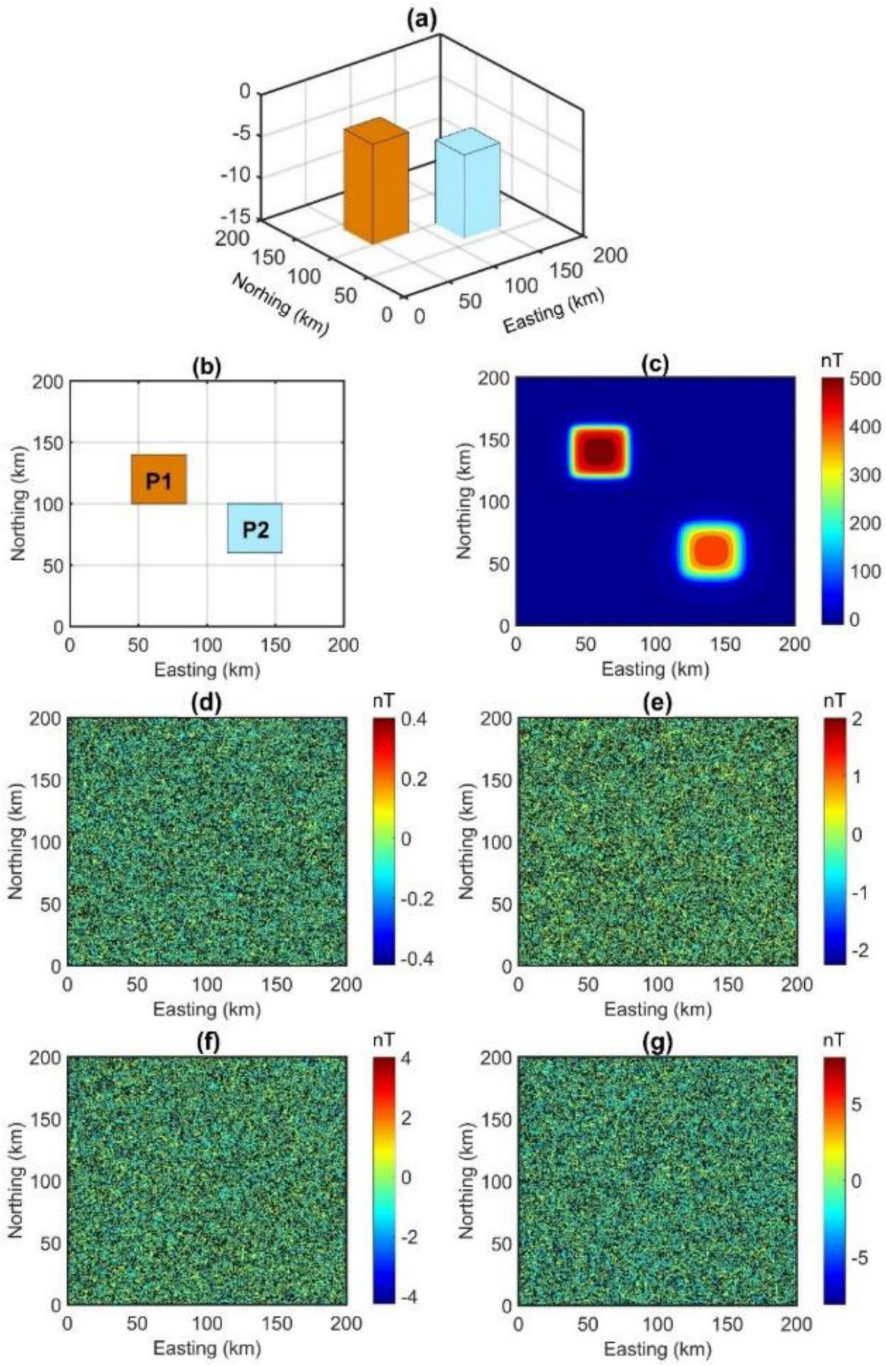
3D view (**a**), plan view (**b**) and magnetic anomaly (**c**) of the model, Gaussian noise with a standard deviations of 0.1 nT (**d**), 0.5 nT (**e**), 1nT (**f**) and 2 nT (**g**).

**Figure 2:**
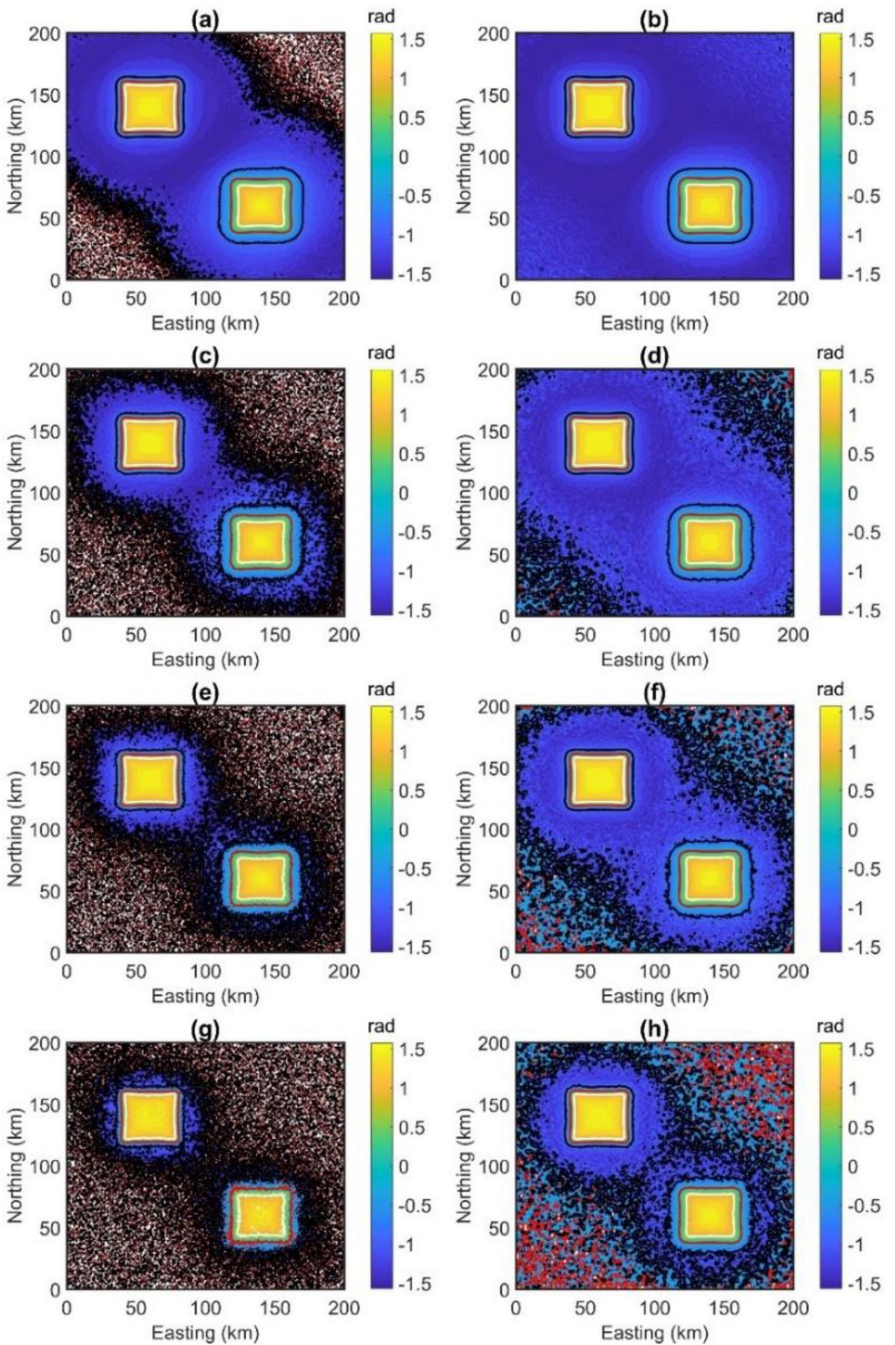
(**a**, **b**) TDR of data with noise in Fig. 1d performed using respectively the frequency domain method and β-VDR method, (**c**, **d**) TDR of data with noise in Fig. 1e performed using respectively the frequency domain method and β-VDR method, (**e**, **f**) TDR of data with noise in Fig. 1f performed using respectively the frequency domain method and β-VDR method, (**g**, **h**) TDR of data with noise in Fig. 1g performed using respectively the frequency domain method and β-VDR method. 0°, 45° and − 45° contours are shown by the red, white line, and black lines.

We designed a synthetic model that includes two prismatic sources located at different depths. Figures 1a and b present 3D and plan views of the sources. The geometric and magnetic parameters of these sources are presented in Table 1. The magnetic anomaly of the model is displayed in Fig. 1c. To simulate the data collected at the field, some authors^43–45^ have added Gaussian noise with a standard deviation of 0.1 nT to their synthetic data. Here, we added Gaussian noise with standard deviation of 0.1 (Fig. 1d), 0.5 (Fig. 1e), 1 (Fig. 1f) and 2 nT (Fig. 1g) to synthetic data before calculating the TDR and the depths.

**Table 1:** Parameters of the model.

Parameters	P1	P2
x-coordinates of center (km)	120	80
y-coordinates of center (km)	65	135
Width (km)	40	40
Length (km)	40	40
Depth (km)	3	6
Declination (°)	0	0
Inclination (°)	90	90
Magnetization (A/m)	− 1	1

From noise-corrupted magnetic data, the TDR values are computed using the FFT vertical derivative and β -VDR vertical derivative. Figure 2a,c,e and g show the TDR maps of magnetic data corrupted with noise in Fig. 1d–g performed by using the FFT vertical derivative, respectively. Figure 2b,d,f and h display the TDR maps of magnetic data with noise in Fig. 1d–g performed by using the β -VDR vertical derivative, respectively. We can see that the TDR maps based on the FFT vertical derivative are dominated by an excessive amplification of the high-frequency noise (Fig. 2a,c,e and g). On the contrary, the β-VDR-TDR responded in a more stable way (Fig. 2b,d,f and h).

**Figure 3:**
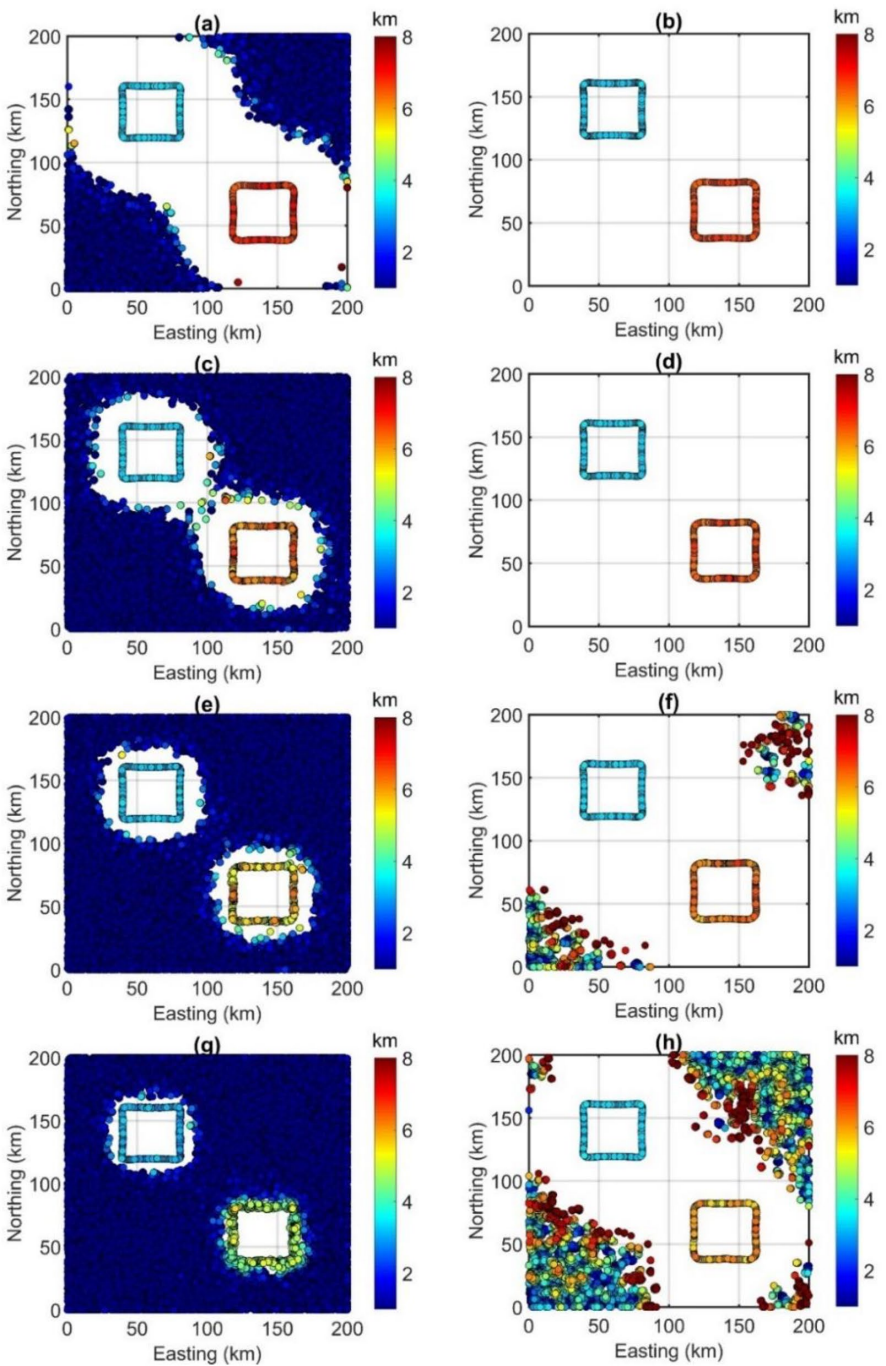
(**a**) FFT-Tilt-depth solutions of data with noise in Fig. 1d, (**b**) β-VDR-Tilt-depth solutions of data with noise in Fig. 1d, (**c**) FFT-Tilt-depth solutions of data with noise in Fig. 1e, (**d**) β-VDR-Tilt-depth solutions of data with noise in Fig. 1e, (**e**) FFT-Tilt-depth solutions of data with noise in Fig. 1f, (**f**) β-VDR-Tilt-depth solutions of data with noise in Fig. 1f, (**g**) FFT-Tilt-depth solutions of data with noise in Fig. 1g, (**h**) β-VDR-Tilt-depth solutions of data with noise in Fig. 1g.

**Figure 4:**
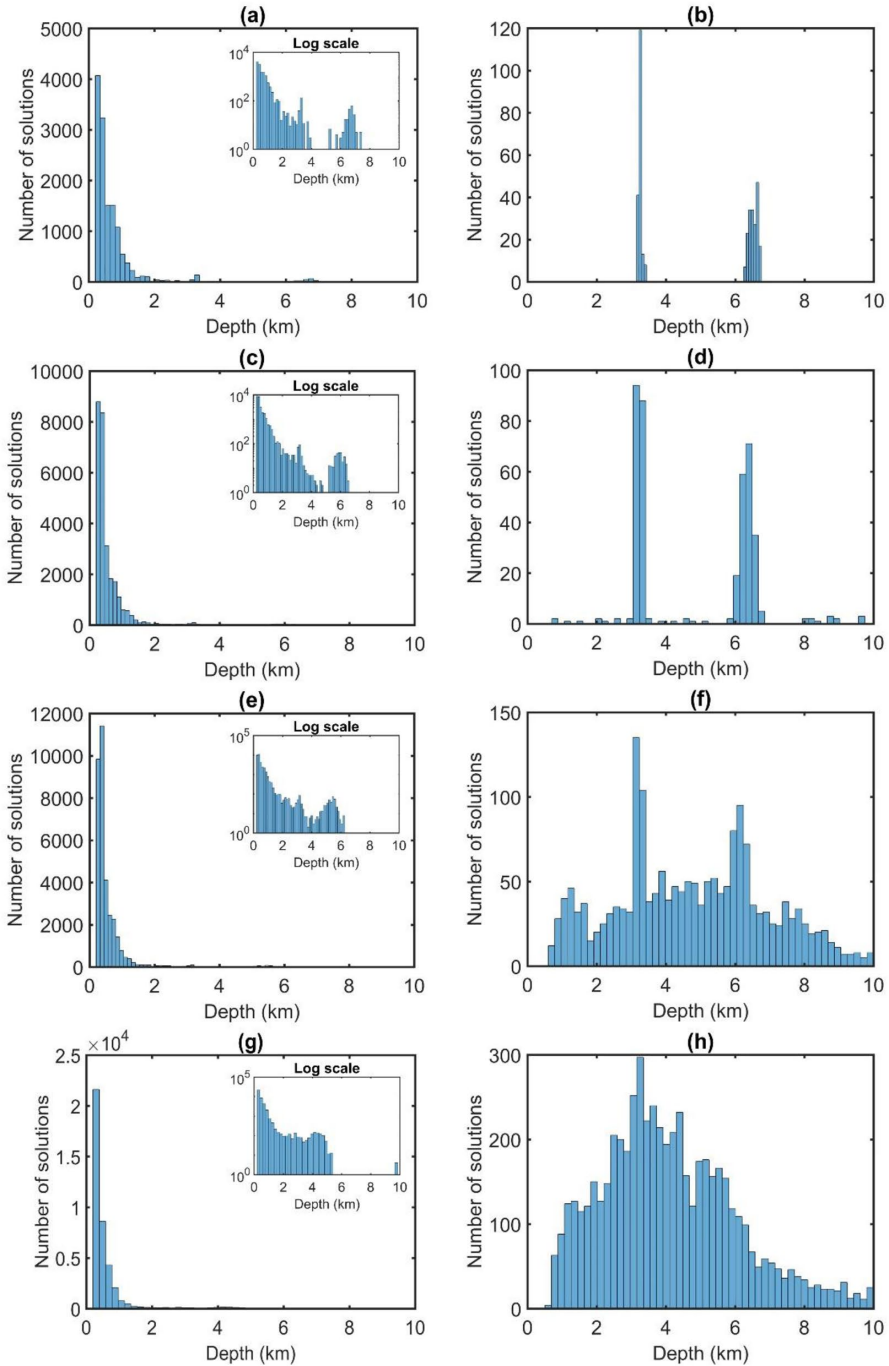
(**a**) Histogram of the depth estimates in Fig. 3a, (**b**) Histogram of the depth estimates in Fig. 3b, (**c**) Histogram of the depth estimates in Fig. 3c, (**d**) Histogram of the depth estimates in Fig. 3d, (**e**) Histogram of the depth estimates in Fig. 3e, (**f**) Histogram of the depth estimates in Fig. 3f. (**g**) Histogram of the depth estimates in Fig. 3g, (**h**) Histogram of the depth estimates in Fig. 3h. Log scale histograms of the depth estimates using the FFT-Tilt-depth were also added to (**a**, **c**, **e** and **g**).

Through the TDR values in Fig. 2, the depths to the sources in magnetic maps are estimated by the FFT-Tilt-depth and β-VDR-Tilt-depth and shown in Fig. 3, and histograms of the depths are shown in Fig. 4. Since the FFT-Tilt-depth brings many solutions, histograms with logarithmic scale are also added to Fig. 4a,c,e and g. Figure 3a and b display the depth solutions of magnetic data with noise in Fig. 1d obtained from the FFT-Tilt-depth and β-VDR-Tilt-depth, respectively. Histograms of the depth estimates in Fig. 3a and b are shown in Fig. 4a and b, respectively. We can see from Figs. 3a and 4a that the FFT-Tilt-depth brings many false solutions in the southwestern and northeastern corners and it overestimates the depth of the deeper source. On the contrary, the β-VDR-Tilt-depth can determine the locations and depths of the bodies much better than the conventional method. Its histogram shows two sets of solutions, one set located above the boundaries of the shallow body P1, and the other above the deeper body P2. Clearly, the locations and depths of the sources mapped by the proposed method are consistent with the real values. Figure 3c and d display the depth solutions of magnetic data corrupted Gaussian noise with a standard deviation of 0.5 nT (Fig. 1e) obtained from the FFT-Tilt-depth and β-VDR-Tilt-depth, respectively. Figure 4c and d show histograms of the depth estimates in Fig. 3c and d, respectively. As can be seen from Figs. 3c and 4c, when the noise level is increased, the FFT-Tilt-depth yields more false solutions around the sources. In this case, the β-VDR-Tilt-depth still can provide the locations and depths of the bodies much better than the FFT-Tilt-depth (Figs. 3d and 4d). Figure 3e and f present the depth solutions of magnetic data corrupted Gaussian noise with a standard deviation of 1 nT (Fig. 2f) determined by the FFT-Tilt-depth and β-VDR-Tilt-depth, respectively. Figure 4e and f depict histograms of the solutions in Fig. 3e and f, respectively. It can be observed from Fig. 3e and 4e that the FFT-Tilt-depth produces shallower depths for the source P2, compared to the true depth of 6 km. Again, this method brings many false solutions. The β-VDR-Tilt-depth still gives better results, although some false solutions appear in the southwestern and northeastern corners (Fig. 3f). Figure 3g and h display the depth solutions of magnetic data corrupted Gaussian noise with a standard deviation of 2 nT (Fig. 2d) determined by the FFT-Tilt-depth and β-VDR-Tilt-depth, respectively. Histograms of the depth solutions in Fig. 3g and h are plotted in Fig. 4g and h, respectively. In this case, the FFT-Tilt-depth also yields very many false solutions and provides shallower depths for the source P2. It is noteworthy that the estimates from the presented technique are closer to the real depth.

### Real example

**Figure 5:**
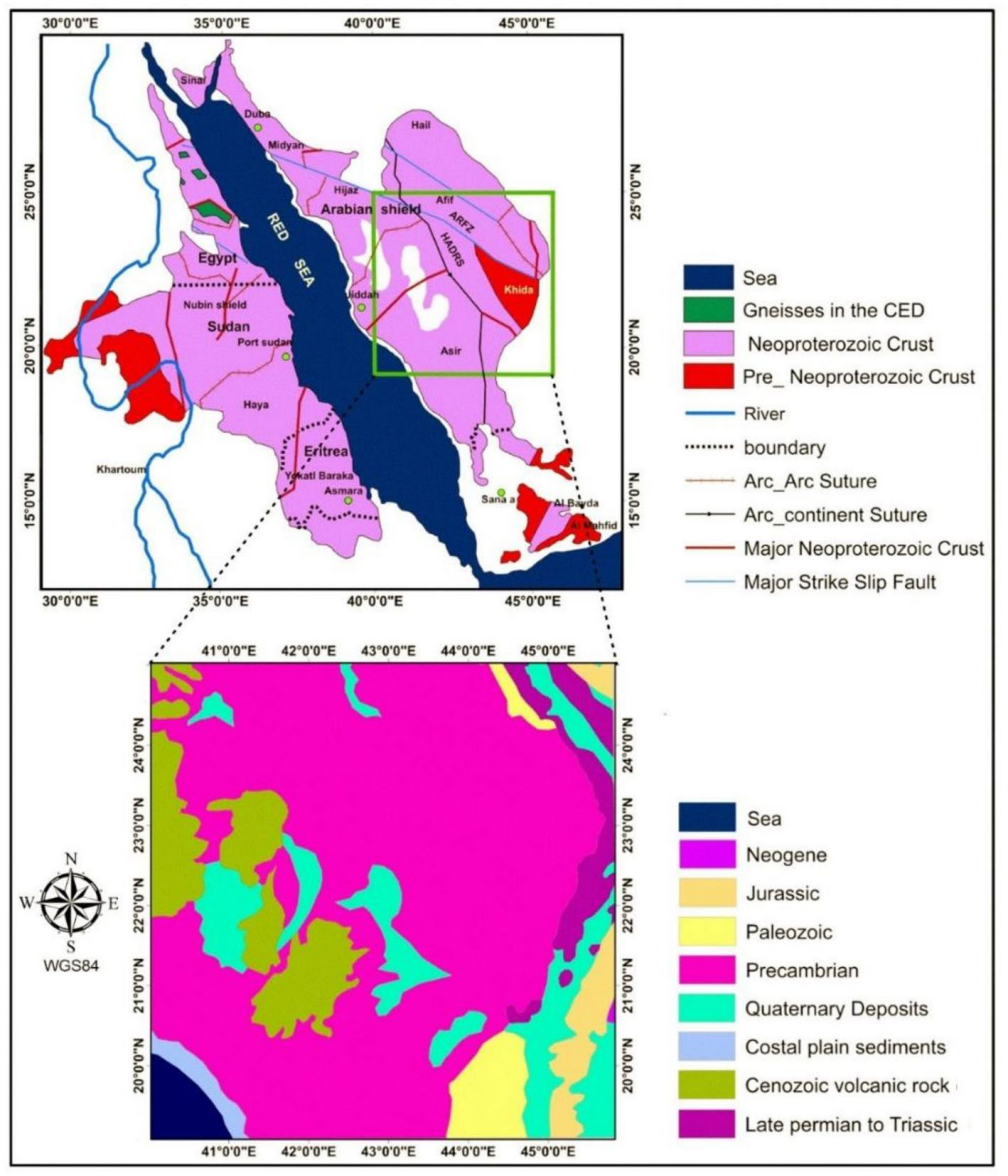
(**a**) Geographic location and geology of the study area (modified from Sahin^46^).

The study area is a part of the Saudi-Arabian Shield (SAS) (Fig. 5). The study part is covered by crystalline rocks that are mostly of Neoproterozoic age^46^. As appeared in Fig. 5, coastal plain sediments are placed along the Red Sea coast to the southwestern corner of the area. Neogene, Jurassic, Paleozoic, and late Permian to Triassic rocks cover the eastern part of the studied area while Wadies and Valleys are filled with Quaternary deposits. Moreover, the western part is intruded by recent Cenozoic rocks (Fig. 5). The SAS contains most industrial and precious metals, including copper, gold, zinc, silver, lead, and tin, which have been prospected in Saudi Arabia in the last 5000 years. Mahd adh Dhahab is the most productive gold abundance in Saudi Arabia^47–49^. Saudi Arabia is distinguished into two specific geologic features: the Saudi-Arabian Shield (SAS) and the Saudi-Arabian (sedimentary rocks) Shelf^50^. The SAS contains both Precamrian-igneous and -metamorphic rocks. Different faults traversed the SAS, such as the Suez-Gulf of NNW-trend and the Najd-fault-system (NFS) of NW^48,51,52^. The eastern part of the SAS is affected by an N–S trend. The southern portion of the SAS is transected by the N–S and NE faults^51^.

The SAS is of economic interest as it incorporates a profitable possibility of metallic ores^49,53^. The significant ore deposits in the SAS are governed by prominent shear zones and subsidiary fault systems^49,54^. Brittle and high-level deformation were interpreted to be linked to ore genesis. The Au-ore genesis is associated with high-grade deformations that were accompanied by substantial fluid discharge^55^. The tectonothermal-deformational events that were occurring during the primal phases of the island-arc forming are controlling the SNS Au-mineralization^49,53,56^. This gives the importance of a stable magnetic interpretation for bringing accurate structures and gives more reliable information about the depths of magnetic sources and the downward extent of the structures that can be interpreted as the pathways of the hydrothermal fluids.

**Figure 6:**
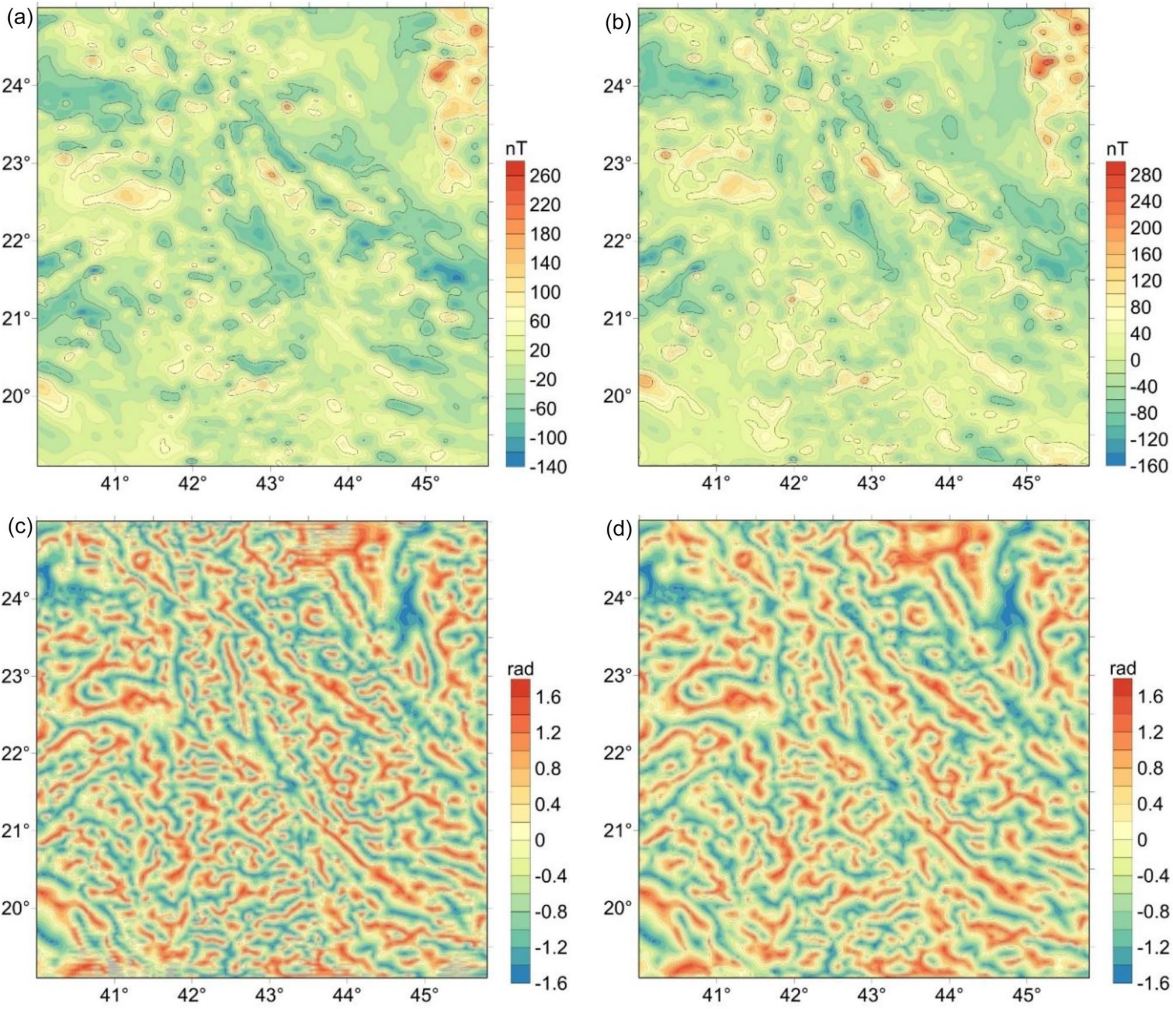
(**a**) Magnetic anomaly of the study area, (**b**) RTP magnetic anomaly, (**c**) Tilt angle of RTP data performed using respectively the frequency domain method, (**d**) Tilt angle of RTP data performed using respectively the β -VDR method.

The magnetic data used in this study were extracted from the EMAG2v3 global magnetic model^57^. The EMAG2v3 data with a resolution of 2 arc-minutes is obtained by a combination of data from satellite, ship, and airborne magnetic measurements. This version is a significant update of the previous release of the Earth magnetic data. Figure 6a depicts the magnetic anomalies of the Arabian Shield. The magnetic inclination and declination of the area are 33.16° and 3.3° respectively, so the reduction to the pole (RTP) of magnetic data using the traditional RTP method^40^ tends to produce unstable results. For this reason, we used a recent method^58^ to overcome the low-latitude problem. Figure 6b depicts the RTP magnetic anomalies of the Arabian Shield. Figure 6c and d show the TDR maps of RTP magnetic data performed by using the FFT vertical derivative and β-VDR vertical derivative, respectively. As can be observed from these figures, the TDR can equalize the amplitudes of large and small anomalies. Both the TDR maps showed that the most prominent structural lineaments observed over the region are in the NW–SE direction.

**Figure 7:**
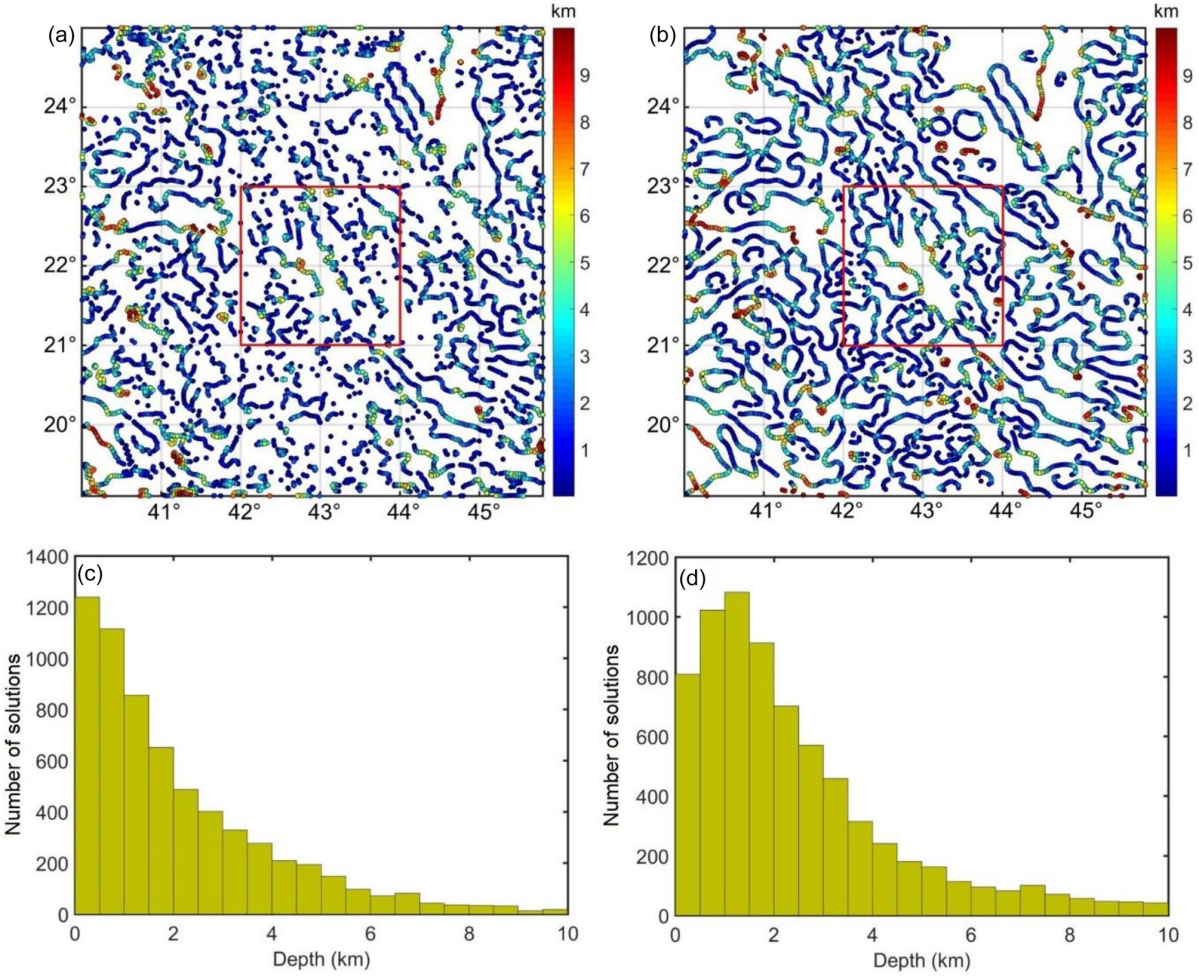
(**a**) FFT-Tilt-depth solutions of RTP data, (**b**) β-VDR-Tilt-depth solutions of RTP data, (**c**) Histogram of the depth estimates in Fig. 6a, (**d**) Histogram of the depth estimates in Fig. 6b.

Figure 7a and b display the depth solutions of RTP magnetic data obtained from the FFT-Tilt-depth and β-VDR-Tilt-depth, respectively. As shown in these figures, the Tilt-depth methods are very useful in highlighting a wide range of structural features of the Arabian Shield and the depths to these structures. The Tilt-depth maps show cleaner geological boundaries compared to the TDR maps. By comparing Fig. 7a and b, we can see that the β-VDR-Tilt-depth allows for better identifications of the magnetic anomalies than the FFT-Tilt-depth. The histograms of the depth solutions obtained from the FFT-Tilt-depth and β-VDR-Tilt-depth are shown in Fig. 7c and d, respectively. One can see that most of magnetic structures in the area exist at 0–6 km depth (Fig. 7c and d).

## Discussion

Unlike the frequency domain technique, the β-VDR formula involves only upward continuation values of magnetic data, which makes the β-VDR method less sensitive to noise. Therefore, the β-VDR method can provide a robust approximation of the vertical derivative of the field. For this reason, the use of the vertical derivative from the β-VDR for the TDR filter allows us to minimize the noise. Quantitative measures of the signal-to-noise ratio in Fig. 2a,c,e and g are 6.2770, 1.1854, − 0.1858, − 1.0451; and in Fig. 2b,d,f and h are 23.8668, 10.4165, 6.7332, 4.1462, respectively. Clearly, in all cases, the FFT-TDR (Fig. 2a,c,e and g) is worse than the β-VDR-TDR (Fig. 2b,d,f and h) with respect to the signal-to-noise ratio. Since the β-VDR-TDR is stable in the presence of noise, it provides clearer images for the source bodies compared to the FFT-TDR. It is noteworthy that, for the highest noise level, the use of the β-VDR-TDR still gives a fairly good result (Fig. 2h).

In this paper, we have suggested using the vertical derivative from the β-VDR for the Tilt-depth to estimate the source edges and depths. The results of the synthetic examples in Fig. 3 and histograms demonstrate that the Tilt-depth method using unstable transformation in the Fourier domain is more affected by the noise than the improved Tilt-depth method by using the vertical derivatives computed from the stable β-VDR method. Figure 3a,c,e and g show strong disturbances in the FFT-Tilt-depth maps. Comparing the results in Fig. 3 and histograms in Fig. 4 demonstrates that the method presented in this paper provides more reliable results for both bodies than the FFT-Tilt-depth.

For the real data case shown in this paper, the delineated linear features in the β-VDR-Tilt-depth are more continuous. The appearance of discontinuous features in the FFT-Tilt-depth map is related to the FFT vertical derivative that is generally unstable in the presence of noise that usually exists in real magnetic data. The FFT-Tilt-depth shows many shallow structures that are located very close to the surface (Fig. 7a and c). As shown in the synthetic studies, these structures may be related to noisy signals. We also note that the use of the β-VDR-Tilt-depth is helpful to remove isolated solutions that are less significant in regional structural studies. Figures 6d and 7b reveal that the NW–SE and WNW-ESE trends are the prominent fault-systems dominating the development of the SAS^48,49^. The results of our new β-VDR-Tilt-depth (Fig. 7b) revealed that the NFS is delineated in near similar streaks extending from SE to NW of the study area^49,52,59^. From the geodynamic point of view, the SAS is one of the Earth’s noteworthy megastructures^60^. Accordingly, the results from the β-VDR-Tilt-depth can be used for interpreting tectonic lineaments and depths of magnetic sources of the SAS and other important megastructures and structurally complicated areas worldwide.

**Figure 8:**
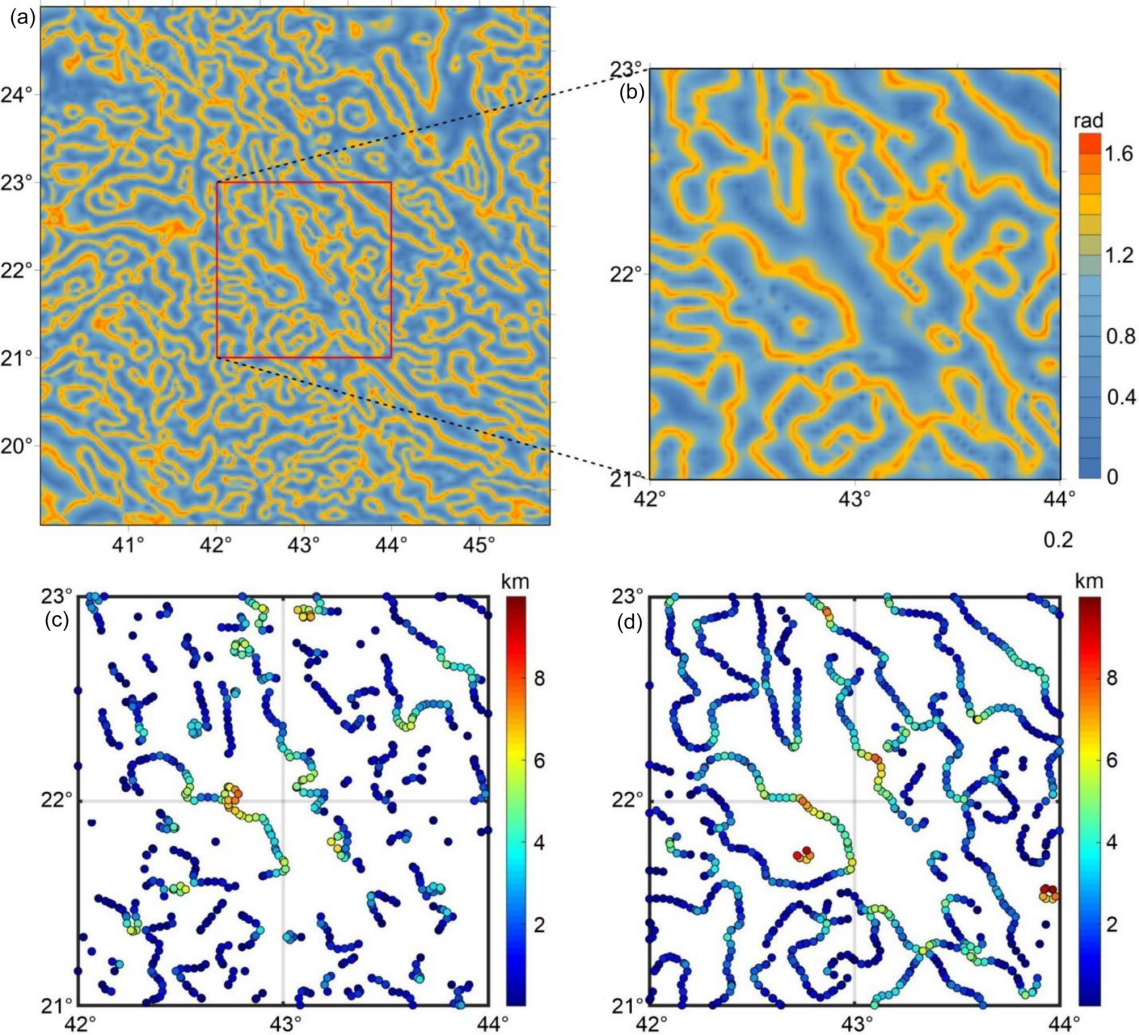
(**a**) TDX of RTP data, (**b**) TDX of RTP data on zoomed area (see red box in Fig. 8a), (**c**) FFT-Tilt-depth solutions of RTP data (**d**) and β-VDR-Tilt-depth solutions of RTP data (**b**) on zoomed area (see red box in Figs. 7a,b and 8a).

To verify the solutions from the presented method, we applied the TDX^38^ to RTP magnetic data to map the structural features of the Arabian Shield. Figure 8a shows the TDX map of RTP magnetic data. It can be seen from Fig. 7b and 8a that the results of the β-VDR-Tilt-depth match well with the structural lineaments extracted by the TDX technique. To better demonstrate the advantages of the β-VDR-Tilt-depth in comparison with the FFT-Tilt-depth, we selected a zoomed area (red box in Figs. 7a,b and 8a) from the study area. The results are shown in Figs. 8c and d. One can see that estimates obtained from both methods have revealed the presence of additional magnetized structures located at depths from 5 to 7 km, which are obscured by the Quaternary deposits in the central region. However, the β-VDR-Tilt-depth map also reveals many magnetic sources that are hidden in the FFT-Tilt-depth map. These sources are also verified by the TDX anomaly in Fig. 8b. The β-VDR-Tilt-depth allows for better estimation of source edges and depths in comparison with FFT-Tilt-depth, making determined magnetic structures more continuous.

## Conclusions

We have improved the Tilt-depth method by using the vertical derivatives computed from the stable β -VDR method to interpret magnetic data. The model studies showed that the β-VDR-Tilt-depth can successfully determine the edges and depths of magnetized structures. The proposed method can identify source locations more clearly and with higher accuracy compared to the FFT-Tilt-depth. In addition, our method is less sensitive to noise and can bring structures more continuous. Further, the estimated magnetic structures of the Arabian Shield using the present method excellently coincide with the structural lineaments extracted by the TDX technique. The results also show that the β-VDR-Tilt-depth method is not only able to yield source edges and depths more clearly and with higher accuracy, but also reveals the presence of many deep structures that are obscured by surface geology.

## Data Availability

Magnetic data can be accessed from https://www.ncei.noaa.gov/.

## Code availability

The code for the β-VDR vertical derivative can obtained from https://github.com/saulopo/betaVDR, while the code for the Tilt-depth is available at https://github.com/fcastro25/GravMagSuite^61^.
